# Citizen-Centered Mobile Health Apps Collecting Individual-Level Spatial Data for Infectious Disease Management: Scoping Review

**DOI:** 10.2196/22594

**Published:** 2020-11-10

**Authors:** Felix Nikolaus Wirth, Marco Johns, Thierry Meurers, Fabian Prasser

**Affiliations:** 1 Berlin Institute of Health Berlin Germany; 2 Charité – Universitätsmedizin Berlin, corporate member of Freie Universität Berlin, Humboldt-Universität zu Berlin, and Berlin Institute of Health Berlin Germany

**Keywords:** pandemic, epidemic, infectious disease management, mobile apps, automated digital contact tracing, mobility tracking, outbreak detection, location-based risk assessment, public health, informatics, app, infectious disease, COVID-19, review

## Abstract

**Background:**

The novel coronavirus SARS-CoV-2 rapidly spread around the world, causing the disease COVID-19. To contain the virus, much hope is placed on participatory surveillance using mobile apps, such as automated digital contact tracing, but broad adoption is an important prerequisite for associated interventions to be effective. Data protection aspects are a critical factor for adoption, and privacy risks of solutions developed often need to be balanced against their functionalities. This is reflected by an intensive discussion in the public and the scientific community about privacy-preserving approaches.

**Objective:**

Our aim is to inform the current discussions and to support the development of solutions providing an optimal balance between privacy protection and pandemic control. To this end, we present a systematic analysis of existing literature on citizen-centered surveillance solutions collecting individual-level spatial data. Our main hypothesis is that there are dependencies between the following dimensions: the use cases supported, the technology used to collect spatial data, the specific diseases focused on, and data protection measures implemented.

**Methods:**

We searched PubMed and IEEE Xplore with a search string combining terms from the area of infectious disease management with terms describing spatial surveillance technologies to identify studies published between 2010 and 2020. After a two-step eligibility assessment process, 27 articles were selected for the final analysis. We collected data on the four dimensions described as well as metadata, which we then analyzed by calculating univariate and bivariate frequency distributions.

**Results:**

We identified four different use cases, which focused on individual surveillance and public health (most common: digital contact tracing). We found that the solutions described were highly specialized, with 89% (24/27) of the articles covering one use case only. Moreover, we identified eight different technologies used for collecting spatial data (most common: GPS receivers) and five different diseases covered (most common: COVID-19). Finally, we also identified six different data protection measures (most common: pseudonymization). As hypothesized, we identified relationships between the dimensions. We found that for highly infectious diseases such as COVID-19 the most common use case was contact tracing, typically based on Bluetooth technology. For managing vector-borne diseases, use cases require absolute positions, which are typically measured using GPS. Absolute spatial locations are also important for further use cases relevant to the management of other infectious diseases.

**Conclusions:**

We see a large potential for future solutions supporting multiple use cases by combining different technologies (eg, Bluetooth and GPS). For this to be successful, however, adequate privacy-protection measures must be implemented. Technologies currently used in this context can probably not offer enough protection. We, therefore, recommend that future solutions should consider the use of modern privacy-enhancing techniques (eg, from the area of secure multiparty computing and differential privacy).

## Introduction

### Background

In December 2019, a novel coronavirus (SARS-CoV-2) appeared and rapidly spread around the world. COVID-19, the disease associated with the virus, can cause severe respiratory illness, is highly transmissible among humans [1], and is often difficult to detect due to asymptomatic courses [2]. The outbreak was declared a Public Health Emergency of International Concern and has implications for global health and economic development alike.

To contain the pandemic, nonpharmaceutical interventions have been implemented on a broad scale as a rapid response. They aim at slowing down or interrupting the infection process by breaking the chain of infections [3]. Examples include individual measures such as social distancing, the wearing of masks, and self-isolation [4] as well as public measures including travel restrictions [5], closure of public institutions and businesses [6], quarantines, and curfews [7]. Many of these measures, however, come with drastic socioeconomic consequences [8]. For example, it has been estimated that the reduction in economic activity resulting from the lockdown will have an impact on the Euro area that is three to four times larger than the impact of the global financial crisis of 2007 and 2008 [9]. Further, it is expected that the pandemic and subsequent lockdowns will lead to mental health problems [10] and excess mortality indirectly related to COVID-19 [11]. Hence, there is considerable pressure to move toward policies that will allow a return of economic and social life to the former state while effectively containing the spread of SARS-CoV-2. This can probably best be achieved by replacing large-scale interventions with local or even individual interventions based on testing, tracing, and targeted quarantine [12,13]. This, however, requires effective means of disease surveillance.

For example, contact tracing is traditionally the manual process of identifying past contacts of a person with an infectious disease [14]. However, manual contact tracing is time and labor intensive, which is a challenge considering the scale of the COVID‑19 pandemic. Thus, many believe that citizen-centered participatory digital tools are required [15]. Most notably, automated digital contact tracing via mobile apps (henceforth simply called contact tracing) is discussed in public media and scientific literature alike [16-20], and according infrastructures have been implemented in several countries. At the time of writing, the COVID Tracing Tracker Project lists more than 40 different apps [21].

An important aspect of automated contact tracing is its reliance on citizen-centered mobile apps collecting individual-level spatial data. The broad adoption of such apps is considered a major prerequisite for associated interventions to be effective [22], and it has been argued that data security and privacy aspects are a critical factor for adoption [22,23]. This is reflected by an intense discussion in the public and the scientific community about privacy-preserving solutions to the contact tracing problem [24,25] and the multitude of established solutions and projects [26-30]. However, often there is a trade-off between privacy risks associated with and the functionalities provided by solutions developed. The German contact tracing app, for example, focuses solely on contacts and builds upon an infrastructure with few central services [31]. As a result, it only supports contact tracing and does not offer any additional functionalities for disease surveillance.

There are, however, several additional use cases that can be implemented with location-based mobile health apps that could help with managing the current and future pandemics. A few unsystematic overviews of technologies currently implemented to manage the COVID-19 pandemic have been published [23,32,33]. For example, Boules and Geraghty [16] have listed several projects: HealthMap, a system that aggregates and maps informal information sources such as online social networks to create risk maps; WorldPop and EpiRis, which use location-based services and other sources of data to model human mobility to predict the spread of COVID-19; and the close contact detector, a solution that uses data on the movement of people to identify individuals with close contact to people who are infected. Other authors suggested the use of digital thermometers, smart watches, or other mobile health devices to remotely monitor vital signs and potential symptoms of at-risk individuals [32,33]. In addition, there are reviews focusing on solutions for health care professionals [34,35]. What is lacking, however, is a structured analysis of important aspects relevant to the technical properties and functionalities of current and future citizen-centered solutions.

### Objectives

The objectives of this work are to inform the current discussions and to support the development of participatory disease surveillance technologies, providing an optimal balance between privacy protection and pandemic control. In our opinion, these objectives are best met by a systematic analysis of existing literature. Our main hypothesis is that there are dependencies between the specific diseases focused on, use cases supported, technology used, and data protection measures implemented. Furthermore, we hypothesize that these dependencies influence the possible design space of solutions. In particular, we focus on the following dimensions:

- *Use case dimension:* Many of the current discussions are focused on technical aspects and their consequences in terms of privacy risks. When considering use cases, the discussion seems to be focused only on contact tracing. Hence, we aim to obtain a broader view of the existing solutions’ functionalities to facilitate the evaluation of novel concepts and to guide the development of future solutions.
- *Technology dimension:* Many recent developments are based on Bluetooth Low Energy (BLE), which is a relatively new technology, and GPS receivers. However, the choice of technology can also impact the functionalities that can be implemented. To potentially broaden the design space for future tools, our objective is to get a broad overview of the technologies that have been used in prior projects and to analyze potential relationships to the use cases that have been implemented.
- *Disease dimension:* There are specific properties of SARS-CoV-2 that have contributed to the characteristic nature of the current pandemic. To determine whether existing solutions can serve as a blueprint for future work, we aim to investigate use cases and technologies in relationship to disease properties such as the path of transmission, infectiousness, and disease-associated symptoms.
- *Data protection dimension:* Finally, privacy protection is seen as an essential factor in current discussions on apps related to COVID-19. To derive a complete picture of data protection measures that can be implemented in this context, we aim to compile an overview of measures implemented in prior projects.

## Methods

We performed a scoping review, as this type of review is best suited to map research activities in a broad and heterogeneous field [36]. Where applicable, we followed the guidelines of the PRISMA-ScR (Preferred Reporting Items for Systematic Reviews and Meta-Analyses extension for scoping reviews) [37]. No review protocol was registered for this study. From the objectives, we derived the following inclusion criteria for articles:

- *Infectious disease management:* Solutions described must be focused on managing epidemics or pandemics of a pathogen, which is communicable directly from human-to-human, such as COVID-19, or transmitted by a vector, such as Dengue.
- *Mobile health apps:* Solutions proposed must be centered around a mobile device or app, which is used for data collection. Articles may describe a concept or an actual implementation. For instance, articles describing methods for modeling population-level disease spread but not covering methods of data collection were not included.
- *Based on individual-level spatial data:* Solutions described must be enabled by collecting individual-level spatial data (individual persons or small groups such as families), either absolute (eg, location determined via GPS) or relative (eg, contacts determined using Bluetooth), either dynamically (eg, regular updates to track movements) or statically (eg, manual entry of living address).
- *Citizen-centered solution:* Data must be collected either automatically or manually by regular citizens using their own devices. In contrast, solutions focused on data collection by professionals within a health care or surveillance context (such as apps for professional contact tracers) were excluded.

An article needed to fulfill all these criteria to be included. Furthermore, all papers were required to be written in English, published between the years 2010 and 2020 (final search performed on June 19, 2020), and scientific peer-reviewed papers containing original work.

We searched both PubMed and IEEE Xplore, as the topic of this review is placed at the intersection of medicine and technology. First, we specified a set of keywords describing the general context. At least one of these keywords needed to be contained in the title of articles checked for eligibility: *epidemic, pandemic, contact tracing, proximity tracing, surveillance,* or *infectious*. Second, we specified a set of keywords covering the complete spectrum of relevant technology. At least one of these keywords needed to be contained in either the title or the abstract of the articles: *mobile, wearables, smartphone, app, cellular network, NFC* (near-field communication)*, barcode, QR* (Quick Response) *code, GPS, Wi-Fi, Bluetooth, RFID* (radio-frequency identification)*, or magnetometer*. Search results were exported as comma-separated values files, harmonized, and imported into a consolidated spreadsheet. In total, we found 1133 articles, of which 646 were identified via PubMed and 487 via IEEE Xplore.

**Figure 1:**
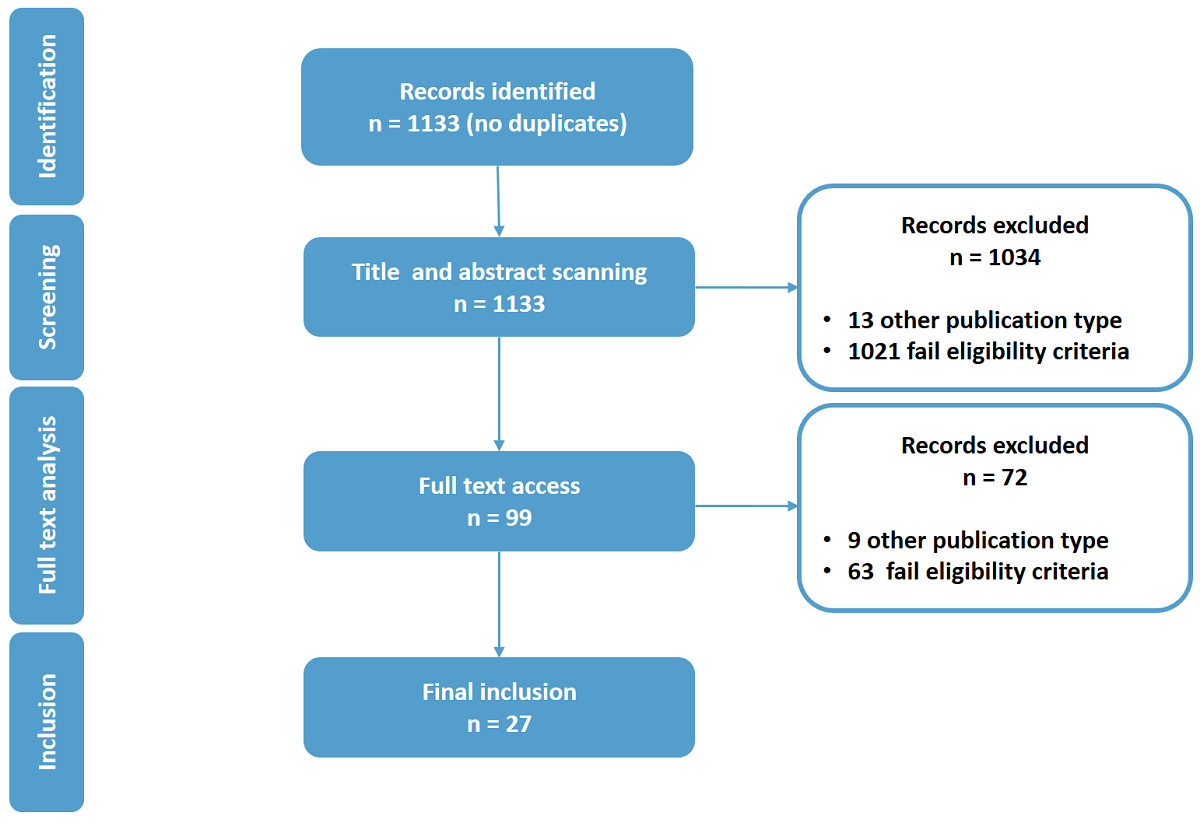
Overview of the selection process.

Figure 1 shows an overview of the selection process. The articles were selected in two steps. In each step, articles were randomly assigned to two authors for assessment, and cases of disagreement were discussed among all authors for consent. In a first screening step, the title and abstract were evaluated regarding the eligibility criteria. Articles were kept if the title or abstract did not provide for a decision on exclusion or inclusion. In total, 99 articles were included after this screening step. Next, the full texts of the selected articles were compared against the eligibility criteria. After this analysis, 27 articles were regarded as finally relevant and were included into the data extraction and analysis process. In this process, we extracted the year and free text for the remaining variables and mutually created categories from the text extracted. The data is available in Multimedia Appendix 1.

Table 1 illustrates the data items collected from the final selection of articles to answer the research questions previously outlined.

**Table 1:** Data items collected.

Variable	Examples	Definition
Year	2010, 2018,...	The year the article describing the solution was published
Use cases	Contact tracing, outbreak detection,...	The infectious disease management processes addressed by the solution
Sensor technologies	GPS, Bluetooth,...	The technology used for spatial tracking
Disease	COVID-19, influenza-like illness,...	The disease the solution focused on
Data protection	Geospatial aggregation, pseudonymization,...	Measures applied to protect the privacy of the users (technical and organizational)

The data collected corresponds with the dimensions investigated. First, we collected the year of publication to analyze developments over time. Second, in accordance with the first dimension, data was collected on the use case or cases supported by the apps. Third, in accordance with the second dimension, the sensor technology used to obtain spatial data was recorded. Fourth, in accordance with the third dimension, we collected data on the diseases that the solutions aimed to manage. Finally, in accordance with the fourth dimension, we recorded the data protection mechanisms used.

During data collection, the spreadsheet document was extended to work as a chartering form containing all data items. In accordance with our research questions, data was then analyzed by calculating frequency distributions of variables’ values and by analyzing the relationships between variables using heat maps, visualizing the combined frequencies of their values.

## Results

### Overview

Table 2 shows the final selection of articles together with the collected data.

**Table 2:** Selected articles and data collected.

Author	Year	Use case	Disease	Sensor technology	Data protection measures
Abbas and Michael [38]	2020	Contact tracing	COVID-19	Bluetooth	Data minimization, pseudonymization
Abeler et al [24]	2020	Contact tracing	COVID-19	Bluetooth	Pseudonymization
Ackley et al [39]	2020	Outbreak detection	ILI^a^	GPS, IP^b^ address geolocation	Geospatial aggregation, pseudonymization, temporal aggregation
Barrat et al [40]	2014	Contact tracing	Generic	Bluetooth	None
Chan et al [41]	2020	Outbreak detection	COVID-19	Manual entry	Consent, data minimization, pseudonymization
Farrahi et al [42]	2014	Contact tracing	Generic	Bluetooth, phone logs	Consent, temporal aggregation
Ferretti et al [43]	2020	Contact tracing	COVID-19	Code scanning, GPS	Consent, transparency
Jeong et al [44]	2019	Contact tracing	Generic	Magnetometer	Geospatial aggregation
Kim et al [45]	2019	Outbreak detection	ILI	Not specified	None
Kim et al [46]	2016	Location-based risk assessment, mobility tracking	Zika	GPS	None
Leal Neto et al [47]	2017	Outbreak detection	Generic	GPS, manual entry	Data minimization
Leal Neto et al [48]	2020	Outbreak detection	Generic	Not specified	Consent
Lwin et al [49]	2014	Outbreak detection	Dengue	GPS, manual entry	None
Michael and Abbas [50]	2020	Contact tracing	COVID-19	Bluetooth	Pseudonymization
Miller et al [51]	2018	Location-based risk assessment, outbreak detection	ILI	GPS	None
Navin et al [52]	2017	Location-based risk assessment, outbreak detection	Generic	Not specified	None
Nguyen et al [53]	2017	Contact tracing	Generic	Magnetometer	Geospatial aggregation
Olson et al [54]	2017	Outbreak detection	Gastroenteritis	Manual entry	None
Okumura [55]	2019	Contact tracing	Generic	GPS, GSM^c^	Data minimization
Prieto et al [56]	2015	Outbreak detection	ILI	Manual entry	None
Rodriguez-Valero et al [57]	2018	Outbreak detection	Zika	GPS	Pseudonymization
Sugiura et al [58]	2010	Outbreak detection	Generic	Manual entry	None
Tripathy et al [59]	2020	Contact tracing	COVID-19	Bluetooth	None
Vazquez-Prokopec et al [60]	2013	Mobility tracking	Generic	GPS	None
Wang et al [61]	2020	Contact tracing	COVID-19	GPS	None
Yasaka et al [20]	2020	Contact tracing	COVID-19	Code scanning	Pseudonymization
Zhang et al [62]	2013	Contact tracing	Generic	Bluetooth	None

^a^ILI: influenza-like illness.

^b^IP: Internet Protocol.

^c^GSM: Global System for Mobile Communications.

As can be seen, more than half of the articles have been published in the last 3 years, indicating a current interest in the topic. In the following sections, we will present further analyses to investigate the questions outlined in the “Objectives” section.

### Use Case Dimension

The first objective of this study was to take a broader look at functionalities of mobile health apps using spatial data for infectious disease management.

Within the selected set of articles, we identified four use cases, which we assigned to two distinct categories. The first category is *user-centered services* (ie, solutions focusing on individual health). The first such use case is *automated contact tracing*, where data on contacts between individuals and on infections is used to determine exposure risk and to notify individuals if necessary. The second such use case is *location-based risk assessment*, where the locations of individuals are used to warn them when entering areas with high disease activity. The latter type of solutions might also be used to estimate individual exposure risk.

The second category is *disease surveillance* (ie, solutions focusing on population health), which involves collecting and analyzing data to monitor the occurrence of diseases within the population with the aim to support public health interventions. The first use cases in this category were *outbreak detection*, which can include syndromic surveillance (eg, based on symptoms reported or data about confirmed cases to determine areas with disease activity). The second such use case is *mobility tracking*, where patterns about the movement of larger groups of individuals are determined to support various analyses, such as the modeling of disease dynamics.

We emphasize that, although these use cases can be implemented independently of one another, there are obvious relationships. For example, data collected via an outbreak detection app could also be used to provide location-based risk assessment services. Moreover, both individual-level as well as population-level use cases ultimately aim to protect the entire population and its individuals. Table 3 illustrates how often the use cases have been described.

**Table 3:** Overview of use cases covered.

Use case	Articles, n
Contact tracing	13
Outbreak detection	12
Location-based risk assessment	3
Mobility tracking	2

Within the 27 papers, automated contact tracing has been addressed 13 (48.1%) times. Outbreak detection was covered by 12 (44.4%) of the papers. Location-based risk assessment was described in 3 (11.1%) papers, and mobility tracking in 2 (7.4%) papers.

As mentioned before, different use cases can also be combined with each other. However, the articles identified are quite specialized: 24 (89%) focused on a single use case. The remaining 3 (11%) all combined location-based risk assessment with an additional use case.

**Figure 2:**
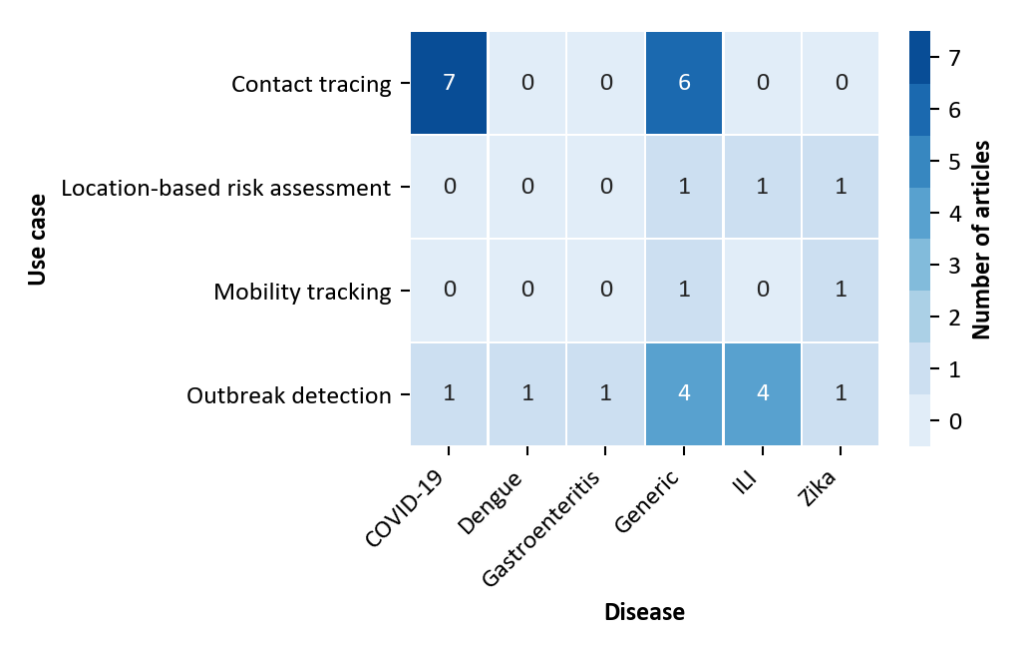
Relationships between diseases and use cases described. ILI: Influenza-like illness.

Current discussions on participatory mobile health solutions in the context of COVID-19 largely focus on automated contact tracing only. To gain insights into potential directions for future developments, we also analyzed which use cases have been described for which disease (see “Disease Dimension” section for more details). Figure 2 shows the common distribution of use cases described and diseases focused on.

As can be seen, in accordance with current discussions, papers that address COVID-19 focused nearly exclusively on automated contact tracing. For other diseases, however, several further use cases have been described. Moreover, a wide range of generic concepts has been proposed independently of a concrete disease. These focused mainly on automated contact tracing and outbreak detection. We also analyzed in which year solutions for certain use cases have been described in the articles analyzed. An interesting finding is that 69% (9/13) of the papers focusing on automated contact tracing were published in the years 2019 and 2020, while 71% (10/14) of all papers focusing on other use cases were published in 2018 or earlier.

### Technology Dimension

In this dimension, we aimed to obtain an overview of technologies that have been proposed to implement the solutions identified. Moreover, we wanted to gather insights into the degree to which technical implementation options and supported functionalities are related to one another.

In total, we identified eight distinct technologies that have been proposed for collecting spatial data: (1) GPS receivers collect absolute coordinates using a satellite system, (2) Bluetooth can be used to estimate the physical proximity between two devices and hence collect relative spatial data (ie, contacts between people), (3) manual entry refers to the manual recording of spatial information such as a living address, (4) magnetometers can be used to measure the small magnetic interferences produced in proximity to a second magnetometer, (5) code scanning refers to the scanning of a code such as a QR code to record a contact or a certain location, (6) Global System for Mobile Communications (GSM) can be used to estimate the absolute geospatial position, (7) Internet Protocol (IP)–based geolocation refers to the estimation of the absolute location by checking the range a user’s IP address is assigned to, and (8) phone logs refer to the relative position between people by analyzing phone calling lists and text messages. The frequency with which individual technologies were used is displayed in Table 4.

**Table 4:** Overview of technologies used for collecting spatial data (three solutions with unspecified technology excluded).

Sensor technology	Articles, n
GPS	10
Bluetooth	7
Manual entry	6
Code scanning	2
Magnetometer	2
Phone logs	1
IP^a^ address geolocation	1
GSM^b^	1

^a^IP: Internet Protocol.

^b^GSM: Global System for Mobile Communications.

Out of 27 articles, GPS, as the most frequent technology, has been described 10 (30.3%) times, followed by Bluetooth, which was mentioned 7 (21.1%) times, and manual entry, which was described in 6 (18.2%) papers. Magnetometers and code scanning were each mentioned in 2 (6.1%) papers. GSM, IP-based geolocation, and phone logs were each suggested in 1 (3.0%) paper. It is noteworthy that 3 articles did not explicitly specify the technology suggested for collecting spatial data (see [45,48,52]). Moreover, it can be noted, that none of the papers described solutions using wireless local area network access points. We also analyzed the ability of the solutions described to track an absolute position (longitude and latitude) or a relative position (proximity to other devices): 60% (n=18) are able to measure absolute positions, while 40% (n=12) can only determine relative positions.

When looking at the time at which papers have been published that use the two most common technologies, GPS and Bluetooth, spikes in the frequency of mentions of Bluetooth can be seen in the years 2013-2014 (the time when the technology first became available on a large scale on commodity devices) and 2020 (the time of the SARS-CoV-2 pandemic). Articles about solutions using GPS have been published continuously over time.

**Figure 3:**
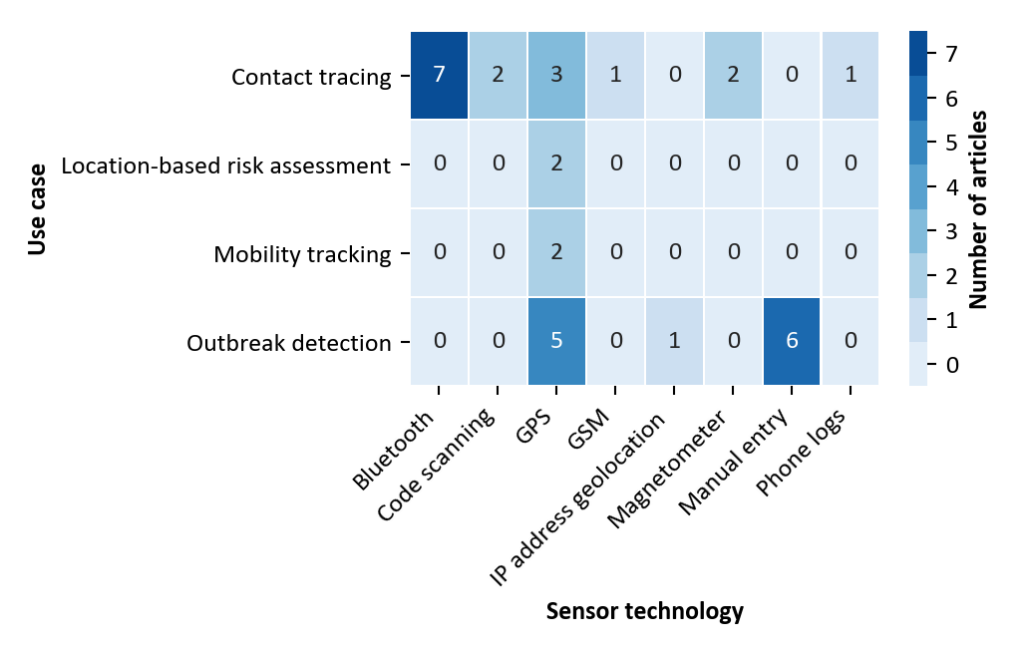
Relationship between use cases and technology used to capture spatial data (three solutions with unspecified technology excluded). GSM: Global System for Mobile Communications; IP: Internet Protocol.

To study how technology might influence the use cases implemented, we analyzed the relationships between both aspects. The result is shown in Figure 3.

It can be seen that Bluetooth is a common technology that has only been used to implement contact tracing (7 papers). Manual data entry is also common but has only been used for outbreak detection (6 papers). GPS, however, is a frequent and versatile technology that has been proposed or implemented for all four use cases identified (12 papers in total). The two remaining technologies that have been suggested more than once are code scanning and magnetometers. Both have only been used to support contact tracing solutions.

### Disease Dimension

An important objective of our analysis was to study potential relationships between disease properties and use cases as well as technologies used. We identified five specific diseases addressed: (1) COVID-19 and (2) influenza-like illness (ILI) are mainly transmitted by droplet infection and cause symptoms like coughing and fever; (3) Dengue and (4) Zika are transmitted mainly by mosquitos and cause symptoms like rash, vomiting, and fever; and (5) gastroenteritis is mainly transmitted by smear infection and causes symptoms like diarrhea, vomiting, and fever. As a first step, we analyzed the frequency with which certain diseases have been addressed by publications. The results are shown in Table 5.

**Table 5:** Overview of diseases targeted by the solutions analyzed.

Disease	Articles, n
Generic	11
COVID-19	8
ILI^a^	4
Zika	2
Dengue	1
Gastroenteritis	1

^a^ILI: influenza-like illness.

Out of 27 articles, 11 (40.7%) of the papers described generic solutions not designed for a specific disease. Of the remaining papers, 8 (29.6%) mentioned COVID-19, 4 (14.8%) mentioned ILI, 2 (7.4%) mentioned Zika, 1 (3.7%) mentioned Dengue, and 1 (3.7%) mentioned gastroenteritis.

**Figure 4:**
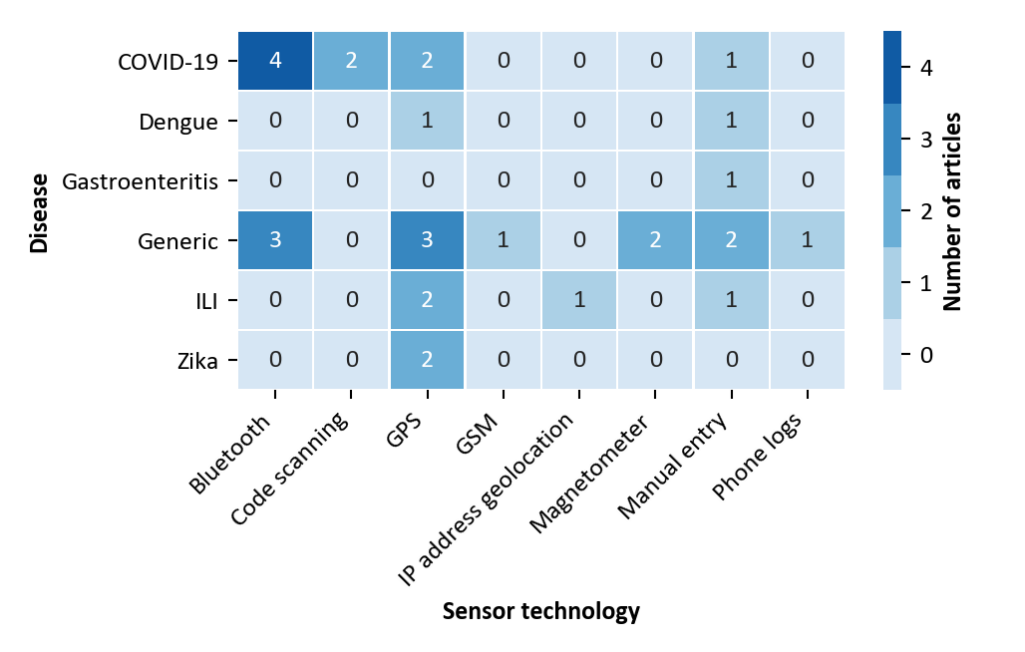
Relationship between diseases and sensor technology (three solutions with unspecified technology excluded). ILI: Influenza-like illness; GSM: Global System for Mobile Communications; IP: Internet Protocol.

We then analyzed whether there was a specific relationship between disease properties and the use cases suggested. For this purpose, we again refer to Figure 3. It can be seen that, obviously, automated contact tracing has only been suggested for highly infectious human-to-human transmissible diseases. For other diseases that are transmitted by vectors, outbreak detection, mobility tracking, and location-based risk assessment are more common. To study whether a similar relationship can also be found regarding the sensor technology, we compiled the data presented in Figure 4.

Figure 4 shows that Bluetooth, which is a solution for exclusively tracking relative spatial data (ie, contacts), has only been suggested for generic disease tracking or for managing COVID-19. In contrast, technologies for tracking absolute positions (primarily GPS) have more frequently been proposed for other diseases. It is also worth mentioning that 2 papers suggested the use of code scanning for COVID-19, which is a versatile technology that can be used to track both absolute and relative spatial data.

### Data Protection Dimension

Data protection is a central aspect in many discussions on automated contact tracing and related use cases. To gain insights into measures that have been proposed, we first analyzed the protection measures mentioned in the selected articles.

We identified six different data protection measures: (1) pseudonymization, which refers to the replacement of identifying information with random meaningless identifiers; (2) geospatial and (3) temporal aggregation, which refers to techniques for reducing the uniqueness of data; (4) data minimization, which is a privacy-by-design measure implying that as little information as necessary is stored and processed; (5) consent, which means that users are explicitly asked to permit data processing, typically within a study setting; and (6) transparency, which refers to the general principle of communicating which data is stored and how it is processed.

Table 2 shows that 52% (n=14) of the papers did list privacy protection measures, while 48% (n=13) of papers did not mention data protection aspects. When taking a look at the time in which the individual papers were published, it can be seen that there is a trend toward more consideration of data protection aspects in recent years. In total, data protection measures were only mentioned in one of the papers published before 2017.

We then analyzed how often the individual measures were suggested or implemented. The results are shown in Table 6.

**Table 6:** Data protection measures mentioned in the articles selected.

Data protection measure	Articles, n
Pseudonymization	7
Consent	4
Data minimization	4
Geospatial aggregation	3
Temporal aggregation	2
Transparency	1

Table 6 shows that pseudonymization, out of 27 articles, has been addressed 7 (33.3%) times, followed by consent and data minimization, which were each mentioned 4 (19.0%) times. Moreover, geospatial aggregation was described 3 (14.2%) times, temporal aggregation 2 (9.5%) times, and transparency 1 (4.7%) time. These measures can be categorized into organizational and technical measures. The organizational measures transparency and consent have been mentioned 5 (23.8%) times, and the remaining technical measures have been mentioned 16 (76.2%) times.

Regarding the relationships between use cases and data protection measures, it must be noted that all 4 use cases identified were potentially privacy-invading. However, data protection measures have not been mentioned in any of the papers covering location-based risk assessment or mobility tracking.

**Figure 5:**
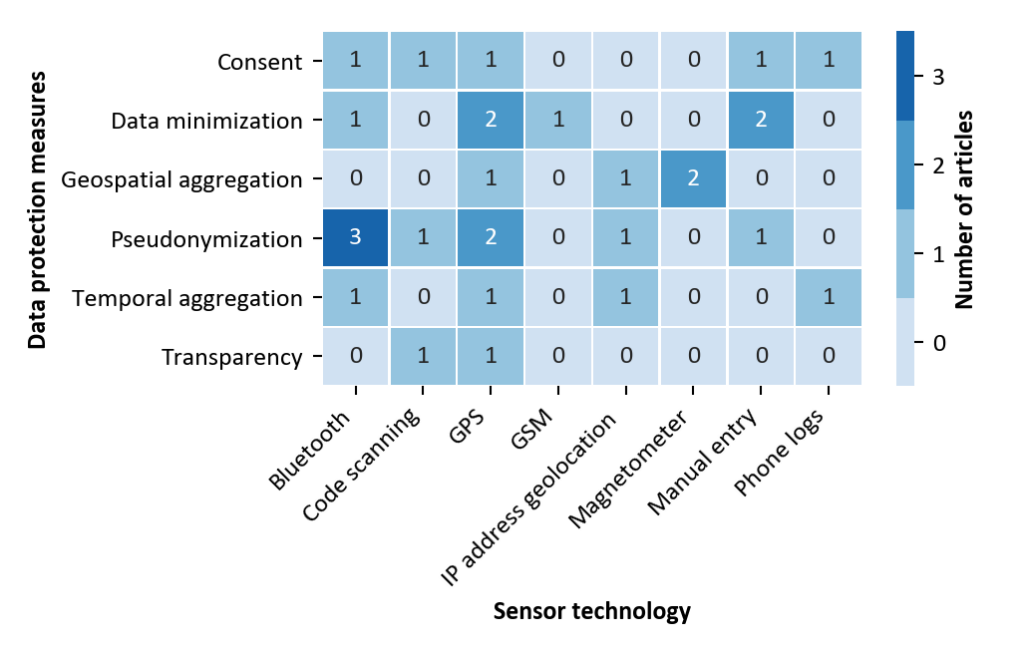
Sensor technologies utilized and protection methods mentioned (three solutions with unspecified technology excluded) GSM: Global System for Mobile Communications; IP: Internet Protocol.

Finally, we also studied the relationship between technologies and protection measures proposed. The results are shown in Figure 5.

It can be seen that many different protection measures have been mentioned in papers focusing on the popular GPS sensor technology. Several measures have also been suggested for protecting Bluetooth-based solutions including pseudonymization, which is particularly important in this context and implemented by exchanging random, pseudonymous identifiers. Most other mentions of protection measures are used in papers focusing on various types of sensors for absolute spatial data other than GPS.

## Discussion

### Use Case Dimension

To broaden the discussion on technical solutions to combat COVID-19 and to propose extension to their functionalities, we first focused on identifying specific use cases. Although most recent publications focus on automated contact tracing, we have identified three additional use cases. Use cases can be grouped into two categories: user-centered services and disease surveillance. All these functionalities have already been discussed and used in the context of COVID-19 [15,16] but in an isolated manner. Future work could, therefore, focus on developing concepts and solutions that support different use cases in a combined manner. Our data also indicates, however, that this might be challenging: 89% (24/27) of all papers analyzed focus exclusively on one use case. Among the notable exceptions is a solution combining outbreak detection with contact tracing for COVID-19 [63].

There might be additional aspects to consider. First, different use cases might be particularly important for different infectious diseases, and the pandemic scale of COVID-19 might be an important factor. We investigated this further in the disease dimension. Second, there might also be technical challenges associated with supporting different use cases and protecting privacy in this process. We investigated this in the technology and data protection dimensions.

### Technology Dimension

We found eight different technologies in the selected literature from which GPS and Bluetooth were most frequently suggested. This is consistent with current discussions on contact tracing apps, which seem to be dominated by BLE. Most technologies were only proposed within the context of a specific use case due to their differences in supporting the measurement of spatial data. For example, Bluetooth has only been proposed for contact tracing, as this technology is only suited for detecting the proximity between individual devices. Moreover, manual data entry has only been suggested for outbreak detection, as it is only suited for tracking static locations such as living addresses. Our results also show that GPS is a highly versatile technology, which has been proposed as a basis for all use cases considered in at least 1 paper. This makes sense, as GPS can be used to collect dynamically absolute locations, which can also be used to determine (to some degree) relative and static spatial data. However, a major challenge arising from this is privacy, which we cover in the data protection dimension.

Our results also suggest that it might be worthwhile to combine different technologies to implement multiple use cases. For example, Bluetooth could be used to implement automated contact tracing, combined with GPS receivers to support location-based risk assessment and mobility tracking. Moreover, this could be combined with manual entry of symptoms or integrated with wearables to collect further data on disease spread. An important prerequisite might be, however, that adequate privacy-enhancing technologies are implemented.

### Disease Dimension

Our results showed that COVID-19 is already the most frequently covered disease in papers on automated contact tracing or related use cases for managing infectious diseases. Obviously, this is due to the scale and global consequences of the COVID-19 pandemic. Moreover, our data shows that most solutions for managing COVID-19 have suggested Bluetooth as the main technology for collecting spatial data. Apart from privacy considerations, this is also well justified by disease properties: COVID-19 is highly infectious, droplet infection is an important route, and there can be a long period of infectiousness before symptom onset. Thus, a technology is needed that can detect close proximity between people at scale with relatively high accuracy [64]. With diseases like Zika and Dengue that are vector-borne, technologies are needed that can capture absolute spatial data (eg, to identify clusters of infections). Code scanning is a technology that can measure absolute and relative spatial data and may, therefore, also be suited for managing diseases like COVID-19. However, code scanning is cumbersome to use, as it is nonautomated and may, therefore, not be well received by the public. It was noticeable that we did not find any articles on several common infectious diseases such as malaria or HIV. The reason is, as we demonstrated, that disease properties do have an influence on the adequacy of solutions to manage them, and solutions focused on these diseases fall out of the scope of this study. One example are apps to estimate the size of local mosquito populations, which are important for managing malaria but typically use special equipment such as mosquito traps [65].

### Data Protection Dimension

Our results show that various types of organizational and technical safeguards have been suggested for protecting privacy when implementing citizen-centered disease surveillance apps. Moreover, more emphasis has been put on this aspect in recent years. Due to the scale of the current pandemic, the topic has become even more important.

There are two use cases that we identified (ie, location-based risk assessment and mobility tracking) for which no protection measures have been proposed in the literature investigated. This is surprising, as both can be considered potentially privacy-invading. Future work will be needed to integrate such use cases into current participatory surveillance apps in a privacy-conscious manner.

Regarding relationships between privacy protection measures and technologies used, we found that more measures have been suggested for technologies that are widely employed and whose sensors provide absolute positions. Our data shows that pseudonymization is an important measure for Bluetooth-based solutions and, thus, especially for the contact tracing use case.

Finally, we would like to add that several techniques have only been mentioned in papers describing concepts and might have been suggested with a rather naïve view on the topic. Most importantly, it is well-known that geospatial as well as temporal aggregation are challenging to implement in a manner offering a high degree of protection [66]. Moreover, even if such an implementation can be developed, it might have significant impacts on the precision of location data [67]. At the same time, solutions focusing on relative spatial data combined with pseudonymization and implemented without continuous data exchange with central services such as the German Corona-Warn-App [31] are considered the current “gold standard” in privacy-preserving automated contact tracing. Future solutions combining relative with absolute spatial data to support further use cases might be built using alternative approaches such as secure multiparty computing protocols [68] and differential privacy [69]. Finally, it is worth mentioning that data protection is not only a technical but also a social challenge, as it is highly connected with public reception and trust.

### Conclusion

In this paper, we have analyzed the literature and discovered several relationships between disease properties, use cases, and technologies. To our knowledge, many of these general dependencies have not been described previously. We, therefore, believe that our results can help with enhancing current solutions for contact tracing and related use cases, and with developing novel, more comprehensive concepts. In addition, the described dependencies could support bottom-up development processes leading to solutions that are more likely to stand the test in real-world implementations. Moreover, we have studied data protection measures that have been suggested and discussed their suitability for different technical environments and use cases. We believe that it will be necessary to employ innovative privacy-enhancing technologies to build comprehensive solutions offering additional functionalities such as population surveillance or individual alerting while maintaining the privacy of citizens.

In the future, when more data on the implementation of solutions in the context of the COVID-19 pandemic will become available, we plan to investigate relationships between their properties along the axes considered in this study and outcomes achieved within specific patient populations.

## Appendix

Multimedia Appendix 1 Selection process and collected data.

## Abbreviations

BLE: Bluetooth Low Energy
GSM: Global System for Mobile Communications
ILI: influenza-like illness
IP: Internet Protocol
NFC: near-field communication
PRISMA-ScR: Preferred Reporting Items for Systematic Reviews and Meta-Analyses extension for scoping reviews
QR: Quick Response
RFID: radio-frequency identification
